# Supplementary material for: Polygalae Radix Oligosaccharide Esters May Relieve Depressive-like Behavior in Rats with Chronic Unpredictable Mild Stress via Modulation of Gut Microbiota
Source: Int J Mol Sci. 2023 Sep 9;24(18):13877. doi: 10.3390/ijms241813877 (PMC10530649; doi:10.3390/ijms241813877)
Supplement: Supplementary file 1 [file ijms-24-13877-s001.zip › ijms-2538794-supplementary.pdf]

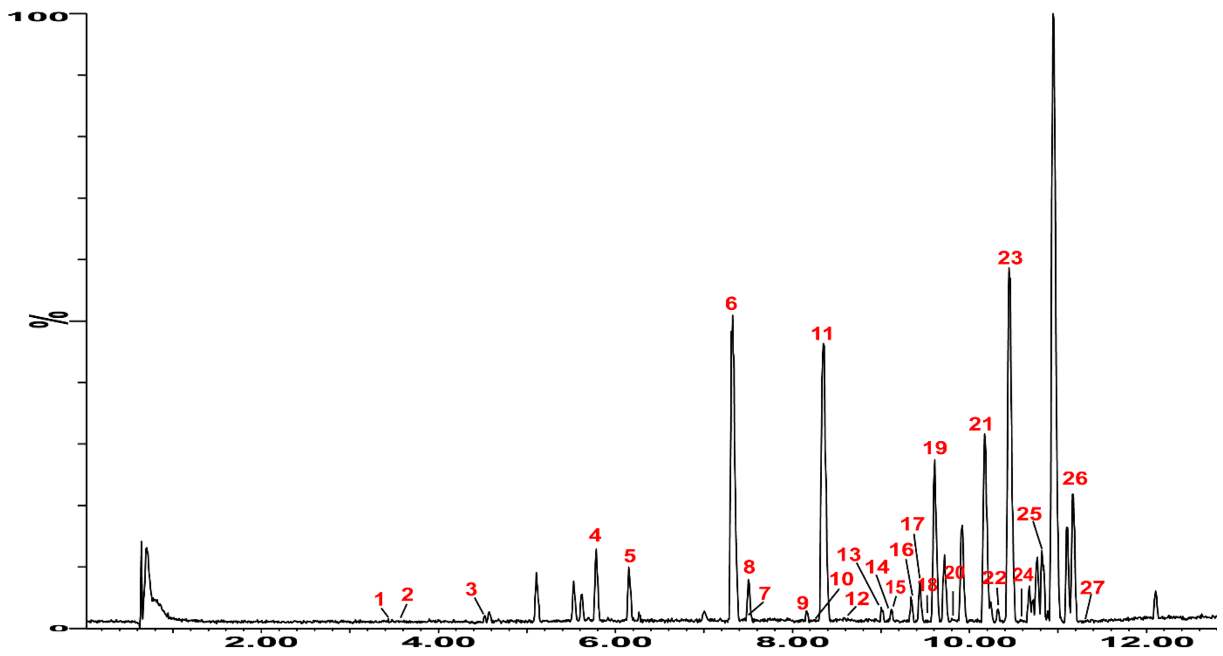

Figure S1 The UPLC-QTOF-MS chromatograms of PROEs

Table S1 UPLC-QTOF-MS data in the negative ion mode of the major chemical components in PROEs

Peak	RT (min)	Molecular formula	Measured (*m/z*)	Calculated (*m/z*)	Mass error /ppm	MS/MS⁻ (–)	Identified compounds
1	3.16	$C_{22}H_{30}O_{14}$	517.1547	517.1563	-3.09	437, 471, 259, 275, 337, 245, 341	Sibiricose A5
2	3.41	$C_{23}H_{32}O_{15}$	547.1660	547.1663	-0.55	205, 473, 223, 341, 271, 367, 275, 283, 471, 437, 431, 495, 531, 337, 429, 317	Sibiricose A6
3	4.29	$C_{23}H_{32}O_{15}$	547.1660	547.1663	-0.55	385, 237, 367, 295, 325, 208, 221, 341, 265, 223, 205	Sibiricose A1
4	5.53	$C_{24}H_{34}O_{15}$	561.1862	561.1819	7.70	337, 287, 217, 369, 340, 281, 499, 209, 265, 279, 307, 365, 517, 529, 345, 221, 473, 323, 443, 251	Sibiricose A2
5	5.94	$C_{30}H_{36}O_{17}$	667.1904	667.1844	8.98	203, 205, 209, 217, 219, 221, 221, 223, 237, 239, 239 245, 247, 263, 265, 277, 281, 295, 299, 305	Tenuifoliside B

6	7.14	$C_{34}H_{42}O_{19}$	753.2262	753.2242	2.65	753, 547, 529, 367, 223, 205	3,6'-Disinapoyl sucrose
7	7.27	$C_{31}H_{38}O_{17}$	681.2036	681.2031	0.73	237, 315, 281, 443, 519, 341, 413, 461, 323, 243, 647, 279, 255, 537, 239, 551, 263, 221, 629, 665	Tenuifoliside A
8	7.31	$C_{33}H_{40}O_{18}$	723.2124	723.2136	-1.66	205, 223, 265, 547, 427, 529, 235, 517, 221, 499, 697, 337, 367, 325, 295, 623, 245, 569, 287, 347	1–Sinapoyl–2–feruloylgentiobiose
9	8.02	$C_{66}H_{84}O_{38}$	1483.4568	1483.4557	0.74	1337, 1161, 1453, 1307, 1119, 1295, 1359, 1275, 1101, 1265, 1143, 1039, 1329, 1317, 1215, 997, 1057, 1099, 1191, 957	Tenuifoliose G
10	8.04	$C_{29}H_{34}O_{15}$	621.1835	621.1819	2.57	499, 517, 235, 485, 571, 223, 295, 413, 481, 447, 307, 273, 323, 277, 217, 263, 247, 347	Reiniose B
11	8.11	$C_{36}H_{44}O_{20}$	681.2036	681.2031	0.73	631, 753, 703, 673, 751, 649, 691, 547, 769, 693, 461, 735, 703, 613, 731, 487, 427, 413, 517, 757	Tenuifoliside A*
12	8.58	$C_{55}H_{68}O_{31}$	1223.3562	1223.3666	-8.50	1077, 955, 809, 1059, 931, 915, 647, 793, 1101, 753, 145, 665, 769, 307, 305, 791, 485, 503, 323, 339	Tenuifoliose S
13	8.85	$C_{56}H_{70}O_{32}$	1253.3682	1253.3772	-7.18	1077, 1161, 955, 1059, 1131, 753, 1119, 1143, 681, 931, 1107, 915, 793, 631, 1039, 1099, 809, 647, 937, 427	Tenuifoliose T

14	8.96	$C_{68}H_{86}O_{39}$	1525.4622	1525.4662	-2.62	1161, 1337, 1323, 1471, 1401, 1143, 1185, 1257, 1319, 1081, 1441, 1119, 1359, 1295, 1039, 1483, 737, 1289, 891, 1061	Tenuifoliose F
15	8.97	$C_{67}H_{84}O_{38}$	1496.4719	1495.4646	5.30	1349, 1203, 1223, 1161, 1337, 1307, 1323, 1143, 1185, 1101, 1257, 1283, 1077, 1107, 1081, 1441, 1119, 1227, 1371, 1295, 1453, 1367, 955, 1039, 1289	Tenuifoliose L
16	9.16	$C_{57}H_{70}O_{32}$	1265.3704	1265.3766	-4.90	1119, 997, 1077, 1101, 955, 1143, 973, 1223, 1059, 851, 835, 753, 1141, 979, 631, 689, 937, 1161, 1099, 735	Tenuifoliose K
17	9.23	$C_{35}H_{44}O_{19}$	767.2399	767.2399	0.00	205, 223, 529, 237, 265, 367, 221, 325, 349, 547, 295, 337, 569, 209, 647, 235, 723, 307, 551, 279	Tenuifoliside C
18	9.34	$C_{56}H_{70}O_{32}$	1253.3682	1253.3772	-7.18	1077, 1161, 955, 1059, 1131, 753, 1119, 1143, 681, 931, 1107, 915, 793, 631, 1039, 1099, 809, 647, 937, 427	Tenuifoliose T*
19	9.43	$C_{58}H_{72}O_{33}$	1295.3794	1295.3878	-6.48	1119, 1077, 997, 973, 851	Tenuifoliose C
20	9.69	$C_{59}H_{74}O_{34}$	1325.3979	1325.3983	-0.30	1149, 1027, 237, 701, 1131, 1203, 728, 1107, 665, 1265, 1225, 1089, 973, 1121, 1009, 273, 987, 1171, 865, 1163	Tenuifoliose P

21	9.73	$C_{59}H_{72}O_{33}$	1307.3835	1307.3872	-2.83	1161, 1039, 1119, 1143, 997, 1185, 1015, 1265, 1101, 1203, 893, 1183, 1021, 795, 979, 877, 673, 1061, 731, 1141	Tenuifoliose I/J
22	9.99	$C_{60}H_{74}O_{34}$	1337.3965	1337.3978	-0.97	1161, 1119, 1295, 1101, 997, 1039, 1143, 1215, 1191, 1173, 1277, 979, 753, 1015, 1149, 1077, 973, 957, 1133, 631, 835, 1021, 851, 1253, 1059	Tenuifoliose B/D
23	10.23	$C_{61}H_{76}O_{35}$	1367.4180	1367.4089	6.65	1191, 1337, 1325, 1069, 1245, 849, 1173, 1119, 1101, 807, 1149, 205, 871, 223, 1295, 1051, 1321, 912, 1029, 829	Tenuifoliose O
24	10.33	$C_{59}H_{72}O_{33}$	1307.3835	1307.3872	-2.83	1161, 1039, 1119, 1143, 997, 1185, 1015, 1265, 1101, 1203, 893, 1183, 1021, 795, 979, 877, 673, 1061, 731, 1141	Tenuifoliose I/J*
25	10.62	$C_{60}H_{74}O_{34}$	1337.3965	1337.3978	-0.97	1161, 1119, 1295, 1101, 997, 1039, 1143, 1215, 1191, 1173, 1277, 979, 753, 1015, 1149, 1077, 973, 957, 1133, 631, 835, 1021, 851, 1253, 1059	Tenuifoliose B/D*
26	11.01	$C_{62}H_{76}O_{35}$	1379.3989	1379.4083	-6.81	1203, 1161, 1337, 1143, 1081, 1257, 1039, 1185, 1319, 1215, 1119, 997, 1021, 795, 1057, 1101, 1175, 1015, 673, 1063, 979, 893, 971, 877, 731	Tenuifoliose A

27	11.14	$C_{63}H_{78}O_{36}$	1409.4067	1409.4189	-8.65	1233, 1337, 1191, 1367, 1173, 1111, 1295, 1287, 1215, 1069, 1349, 1277, 1309, 1245, 1267, 1051, 1057, 1093, 1081, 999, 1149, 1015, 1347, 1213, 1039	Tenuifoliose N

Note *: isomers substances
